# Adjunctive traditional Chinese medicine for ischemic stroke: a systematic review with structured narrative synthesis and GRADE assessment of randomized controlled trials

**DOI:** 10.3389/fmed.2026.1884399

**Published:** 2026-09-04

**Authors:** Ting Liu, Mingjing Lu, Ying Wang, Hailing Ding, Xiangqian Kong

**Affiliations:** 1 Medical College, Qilu Institute of Technology, Jinan, Shandong, China; 2 Department of Traditional Chinese Medicine, Shandong Provincial Hospital Affiliated to Shandong First Medical University, Jinan, Shandong, China

**Keywords:** GRADE, ischemic stroke, modified Rankin Scale, randomized controlled trial, SWiM, systematic review, traditional Chinese medicine

## Abstract

Ischemic stroke is a leading cause of death and long-term disability, and traditional Chinese medicine (TCM) is widely used alongside conventional care in East Asia. We reviewed randomized controlled trials (RCTs) of adjunctive TCM in ischemic stroke, analyzing the evidence by clinical phase and by individual formulation rather than as a single treatment class. We searched seven bibliographic databases and one clinical trial registry to 8 April 2026, for trials of adults with imaging-confirmed ischemic stroke given a TCM formulation in addition to standard care, compared with placebo plus standard care or standard care alone. Nine RCTs enrolling 12,348 randomized participants were eligible. They evaluated eight formulations across three clinical phases, with different outcome instruments and follow-up of 14 days to 24 months, so we did not pool them; each trial is reported with its published effect measure and synthesized under Synthesis Without Meta-analysis guidance. In acute treatment, Tongxinluo improved a favorable functional outcome at 90 days (modified Rankin Scale [mRS] 0 to 1: 640/973 vs. 575/973; odds ratio [OR] 1.33, 95% confidence interval [CI] 1.11 to 1.60), and Panax notoginseng saponins improved functional independence at 3 months (mRS 0 to 2: 1,328/1,487 vs. 1,218/1,479; OR 1.95, 95% CI 1.56 to 2.44); these thresholds are related but not interchangeable. Angong Niuhuang Pills and NeuroAiD (MLC601) showed no clear difference, and a small trial of Xueshuan Xinmai tablets reported no between-group estimate. In early recovery, Qizhitongluo improved lower-limb motor function at 12 weeks (mean difference 1.81 points, 95% CI 0.88 to 2.74). In secondary prevention, Naoxintong reduced 2-year recurrence [hazard ratio (HR) 0.665, 95% CI 0.492 to 0.899] and Dengzhan Shengmai reduced 1-year stroke incidence (HR 0.70, 95% CI 0.50 to 0.98). GRADE certainty, rated separately by formulation, clinical phase, and outcome, ranged from moderate to very low. Safety reporting was incomplete, so comparative safety cannot be established. The review was not prospectively registered and no dated protocol preceded screening. The findings are formulation-specific, unreplicated, and do not support routine clinical use or any conclusion about TCM as a treatment class.

## Introduction

Stroke is a leading cause of death and acquired disability worldwide. In 2021, the Global Burden of Disease Study estimated 11.9 million incident strokes and 7.3 million stroke deaths worldwide, with ischemic stroke accounting for 65.3% of incident cases (1). Survivors frequently retain neurological deficits carrying substantial personal, social, and economic costs, and the burden falls disproportionately on low-income and middle-income countries undergoing rapid demographic transition (1).

Acute stroke care has advanced with intravenous thrombolysis and endovascular thrombectomy (2), but these reach only a minority of patients because of narrow therapeutic windows, strict eligibility criteria, infrastructure requirements, and variable access (3). Many patients treated in time still have residual deficits, and recovery-oriented and preventive therapies are needed to address that gap (4). Traditional Chinese medicine is used alongside contemporary stroke care throughout East Asia, particularly in China, as single herbs, patent medicines, injections and multi-component prescriptions (5, 6).

Proposed mechanisms include neuroprotection and effects on post-ischemic neuroinflammation, oxidative stress, endothelial injury and cerebral microcirculation (7). Several formulations have been tested in adequately sized randomized trials, including Tongxinluo (8), Panax notoginseng saponins in the PANDA trial and its pre-specified secondary analysis (9, 10), Naoxintong (11), Dengzhan Shengmai (12), Qizhitongluo (13) and NeuroAiD (14, 15). These trials differ in more than formulation. They enroll patients at different points after stroke, from within 36 h of onset to as long as 6 months afterward, and measure different outcomes over follow-up periods ranging from 2 weeks to 2 years. Previous reviews have noted that the principal limitation of this literature is methodological rather than quantitative (16).

Two consequences follow. Evidence about one formulation cannot be extrapolated to traditional Chinese medicine as a whole, and evidence generated in the acute phase cannot be extrapolated to rehabilitation or secondary prevention. When trials differ this widely in population, intervention, comparator, outcome, and follow-up, combining them into a single summary estimate yields a number that no clinician or guideline developer could act on. Synthesis Without Meta-analysis (SWiM) guidance was developed for this situation (17), and GRADE allows certainty to be rated transparently for each outcome whether or not pooling is possible (18).

We therefore conducted a systematic review reported with reference to PRISMA 2020 (19) and PRISMA-S (20), with structured narrative synthesis under SWiM, risk-of-bias assessment using RoB 2 (21), and GRADE certainty rating. Our objectives were to characterize the contemporary randomized evidence, to present each trial using the effect measure it published, to organize the synthesis by clinical phase and formulation, and to summarize certainty of evidence and methodological priorities for future trials. We did not undertake a meta-analysis, and we make no claim about traditional Chinese medicine as a unified treatment class.

## Methods

### Protocol, registration and reporting

This review was not prospectively registered in PROSPERO or any other prospective register, and no dated written protocol or statistical analysis plan was completed before screening began. The consequence is specific: readers cannot verify independently that the eligibility criteria, outcomes of interest and analytic approach were fixed before the results of the individual trials were known, and *post hoc* modification of those decisions or selective emphasis among outcomes cannot be excluded. The review has not been registered retrospectively, because retrospective registration would not remedy this limitation and could be mistaken for prospective registration. Accordingly, no analysis reported here is described as pre-specified and no decision is described as having been made in advance. PRISMA 2020 (19) and PRISMA-S (20) were used as reporting frameworks. Items that could not be completed because the original records were unavailable are identified explicitly in the manuscript and Supplementary material. The narrative synthesis follows the SWiM reporting guideline (17). GRADE was used to rate certainty by outcome, formulation and clinical phase (18).

### Eligibility criteria (PICOS)

#### Population

Adults with ischemic stroke confirmed by computed tomography or magnetic resonance imaging. Trials of intracerebral hemorrhage, subarachnoid hemorrhage or transient ischemic attack without imaging-confirmed infarction were not eligible. Trials enrolling mixed stroke populations were eligible only if ischemic subgroup results were reported separately.

#### Intervention

Any traditional Chinese medicine formulation given in addition to conventional stroke care, including single herbs, classical compound prescriptions, Chinese patent medicines and proprietary multi-component preparations. Oral, nasogastric and intravenous routes were eligible. Acupuncture, moxibustion, tuina and other non-pharmacological TCM modalities were not eligible, because the review question concerns pharmacological adjuncts.

#### Comparator

Placebo plus standard care, or standard care alone. Standard care means guideline-concordant management of acute ischemic stroke or of secondary prevention as defined by the guidelines in force at each trial site and time, comprising antiplatelet or anticoagulant therapy as indicated, lipid-lowering therapy, management of blood pressure and glucose, and reperfusion therapy where eligible and available. Trials in which both arms received a traditional Chinese medicine preparation, with no placebo or standard-care-only comparator, were not eligible, because such trials address the choice between TCM regimens rather than the effect of adding TCM to standard care.

#### Outcomes

Functional status, neurological deficit, activities of daily living, motor recovery, recurrent vascular events, mortality and adverse events. Trials whose only outcomes were biomarkers or imaging measures were eligible but are reported separately, because such outcomes are indirect for the clinical questions of interest.

#### Study design

Randomized controlled trials. Non-randomized studies, single-arm studies, protocol publications without results, and observational extension studies of randomized trials were not eligible. Where a trial generated more than one publication, the report of its primary outcome was the primary reference and secondary analyses were used only for supporting detail.

All clinical phases were eligible, a deliberate decision taken to characterize the breadth of the randomized evidence. Because acute treatment, early rehabilitation and secondary prevention are different clinical questions, the synthesis is stratified by clinical phase throughout and no result is combined across phases. Where a trial included more than two arms, only the comparison of the TCM formulation against placebo or standard care contributes to the synthesis.

### Information sources and search

We searched seven bibliographic databases (PubMed, Embase, Web of Science, the Cochrane Central Register of Controlled Trials, China National Knowledge Infrastructure, Wanfang Data and SinoMed) and one clinical trial registry (the Chinese Clinical Trial Registry) to 8 April 2026. MEDLINE content was searched through the PubMed interface and is not counted as a separate database. The reference lists of relevant reviews and of included reports were screened. No language restriction was applied.

The original database-specific search histories and exact executed strings were not retained in the files available for this revision. Accordingly, the search cannot be reproduced exactly from the present record. The databases, registry, final search date and broad concepts are reported, but no retrospectively reconstructed syntax is presented as if it were the search originally run. This limitation is considered when interpreting the completeness of the review. Further information on the searched sources and the status of the unavailable execution details is provided in Supplementary Appendix 1.

Zotero and Rayyan were used for reference management and screening, respectively. Exact software versions were not retained and are therefore not reported.

### Selection and data collection

Records from all sources were imported into reference-management software and deduplicated. Titles and abstracts were screened against the eligibility criteria, and records passing that stage were retrieved in full text and assessed against the same criteria. Data were extracted into a structured form covering study design and setting, participant eligibility and baseline characteristics, intervention composition, dose, route, frequency and duration, comparator and background care, follow-up, primary and secondary outcomes, analysis populations, effect estimates, adverse events, funding and trial registration. Deduplication, title and abstract screening, full-text screening and data extraction are reported as four separate processes.

The surviving review records did not permit independent confirmation of whether title-and-abstract screening, full-text screening and data extraction were each completed in duplicate. The manuscript therefore does not claim duplicate review for these stages. This loss of process documentation reduces auditability and is acknowledged as a limitation. The data fields and figure-level evidence dataset are provided in Supplementary Data File 1.

### Risk of bias

**Figure 1:**
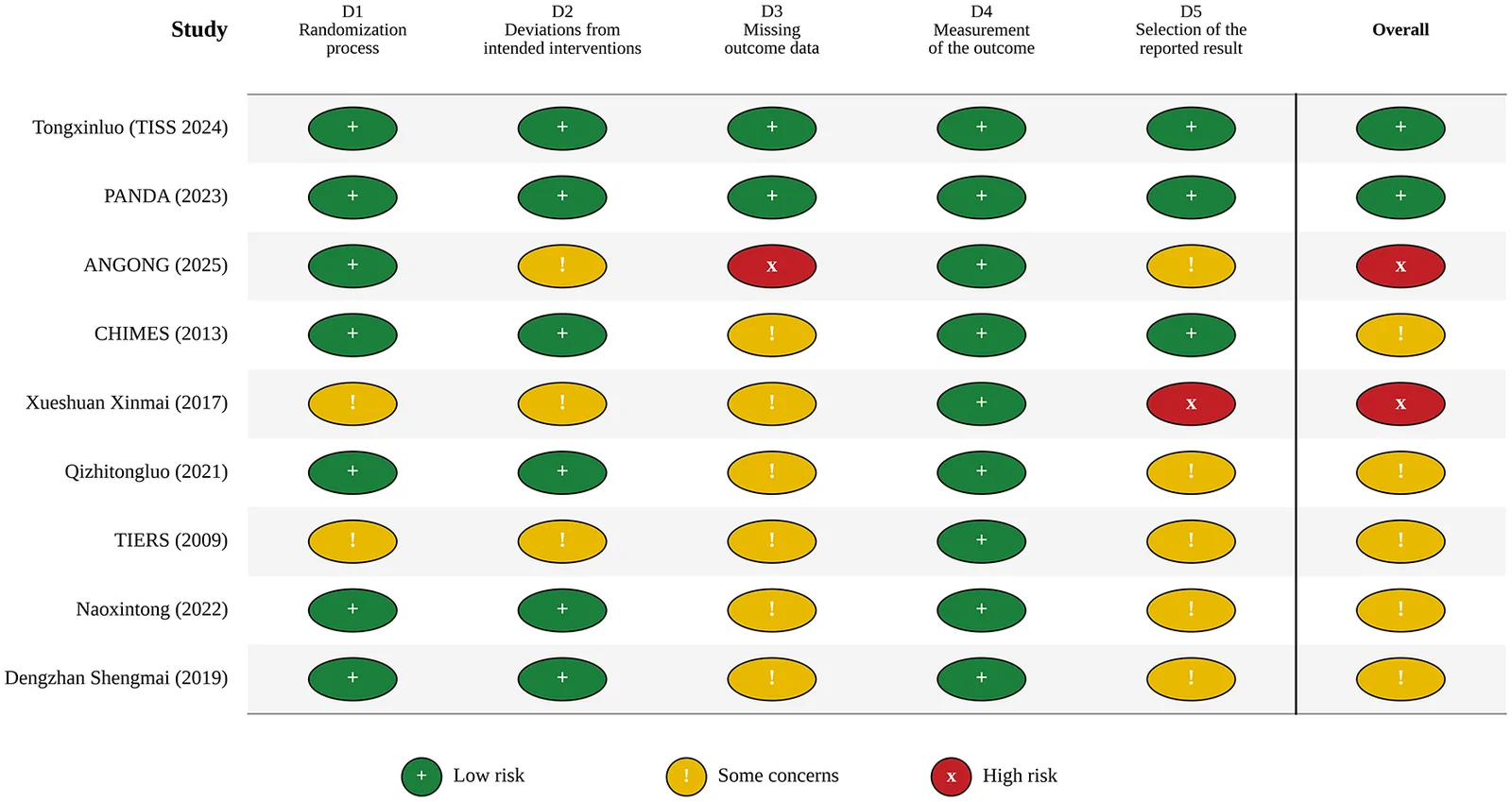
Risk of bias for the specified result in each of the nine included randomized controlled trials, assessed with the Cochrane RoB 2 tool. The specified result is the effect of assignment to the intervention on each trial's primary outcome, or, where no primary outcome was designated, on the most clinically relevant reported outcome. Domains: D1, randomization process; D2, deviations from intended interventions; D3, missing outcome data; D4, measurement of the outcome; D5, selection of the reported result. Overall judgments follow the RoB 2 algorithm. Domain-level rationales and supporting source text are in Supplementary Table 2.

Risk of bias was assessed with RoB 2 (21) for one explicitly specified result in each trial, namely the effect of assignment to the intervention on that trial's primary outcome. Where a trial did not designate a primary outcome, the most clinically relevant reported outcome was selected and identified as the specified result. Assessments used the official signaling questions and algorithm across the five domains, and the overall judgment follows the algorithm, so that it is never more favorable than the least favorable contributing domain. Domain judgments, rationales and supporting source text are in Supplementary Table 2, and Figure 1 and Table 1 are generated from that single dataset.

**Table 1:** RoB 2 judgments for the specified result in each included trial.

Trial (specified result)	D1 randomization	D2 deviations	D3 missing data	D4 measurement	D5 selective reporting	Overall
Tongxinluo (mRS 0-1 at 90 d)	Low	Low	Low	Low	Low	Low
PANDA (mRS 0-2 at 3 mo)	Low	Low	Low	Low	Low	Low
ANGONG (infarct and edema volume at 14 d)	Low	Some concerns	High	Low	Some concerns	High
CHIMES (mRS shift at 3 mo)	Low	Low	Some concerns	Low	Low	Some concerns
Xueshuan Xinmai (NIHSS at 2 wk)	Some concerns	Some concerns	Some concerns	Low	High	High
Qizhitongluo (FMMS-LL at 12 wk)	Low	Low	Some concerns	Low	Some concerns	Some concerns
TIERS (Fugl-Meyer at 4 and 8 wk)	Some concerns	Some concerns	Some concerns	Low	Some concerns	Some concerns
Naoxintong (recurrence at 2 y)	Low	Low	Some concerns	Low	Some concerns	Some concerns
Dengzhan Shengmai (stroke at 1 y)	Low	Low	Some concerns	Low	Some concerns	Some concerns

Assessments relate to the effect of assignment to the intervention on each trial's primary outcome, or, for Xueshuan Xinmai, on the most clinically relevant reported outcome, since that report designates no primary outcome. Overall judgments follow the RoB 2 algorithm and are never more favorable than the least favorable contributing domain. Domain rationales with supporting source text are in Supplementary Table 2. This table, Figure 1 and the GRADE profiles are generated from a single master dataset.

Missing information was handled within the domain to which it belongs. Absence of a reported detail was not treated as automatic evidence of high risk, and neither was it treated as reassurance; where a signaling question could not be answered from the report, protocol or registry entry, the domain was judged to raise some concerns.

Risk-of-bias judgments were reassessed during revision from the published reports, available protocols and registry records and were checked against the RoB 2 algorithm. The available documentation did not establish an independent duplicate assessment; the judgments should therefore be interpreted as a transparent reassessment rather than a documented consensus of two independent assessors.

### Synthesis and certainty of evidence

We did not perform a meta-analysis and report no pooled summary estimate. The decision rests on clinical and methodological differences between the trials, not on data availability. The nine included trials evaluate eight formulations at different doses and routes, started between 36 h and 6 months after onset, assessed with different primary outcome instruments over 14 days to 24 months. Two trials reporting a binary functional outcome at approximately 3 months do so at different thresholds, mRS 0 to 1 and mRS 0 to 2, which are related but not interchangeable. We note explicitly that seven of the nine trials report a primary outcome with a point estimate and confidence interval, so data availability was not the constraint.

Synthesis followed SWiM guidance (17). Trials were grouped first by clinical phase, defined by the interval between onset and start of the study intervention together with the trial's stated aim, then by formulation. For each trial we report the analysis population, the primary outcome, the effect estimate published by that trial with its confidence interval, and, where event counts permit, absolute event proportions. The metric used to classify each result was the direction and precision of that trial's own primary outcome, not a count of statistically significant findings. Results were placed in one of three categories: benefit on the primary outcome, where the published estimate favored the intervention and its confidence interval excluded the null; no clear difference, where the interval included the null; and too incompletely reported to classify, where no between-group effect estimate for a clinical outcome was reported. Secondary outcomes and subgroup findings were not used to reclassify a trial, and vote counting was not used. Where a trial reported an odds ratio or hazard ratio, that measure is reported here; no published odds ratio has been converted to a risk ratio. Any absolute risk difference we calculated from published event counts is identified as such at the point of use.

Certainty was rated using GRADE (18) for each clinically important outcome, separately by formulation and clinical phase, beginning at high for randomized evidence and downgraded for risk of bias, inconsistency, indirectness, imprecision and publication bias where warranted. Because every outcome is informed by a single trial, inconsistency could not be assessed and is recorded as not assessable rather than as absence of concern. Publication bias could not be assessed formally, because each formulation-outcome comparison was generally represented by a single trial. Qualitative concerns remain and are described in the Discussion, but no outcome was downgraded for publication bias, because the available evidence was insufficient to support a defensible outcome-specific judgment. The predominance of trials conducted in China is discussed under indirectness and generalizability rather than as evidence of publication bias, since geography alone does not establish selective publication.

## Results

### Study selection

**Figure 2:**
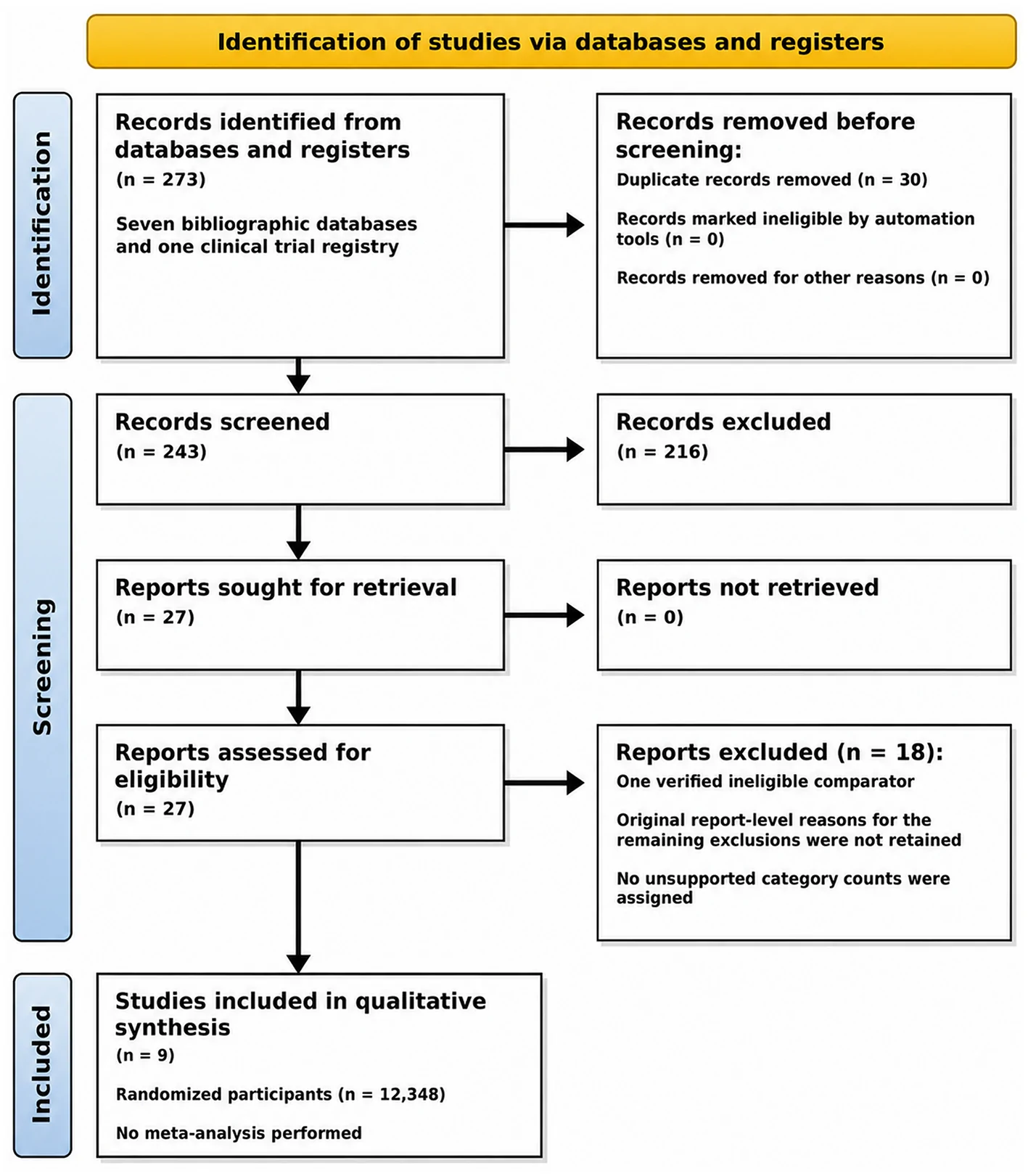
PRISMA 2020 flow diagram of study selection. Of 273 records identified through database and registry searching, 30 duplicates were removed, and 243 records were screened by title and abstract. After 216 were excluded, 27 full-text reports were assessed for eligibility, of which 18 were excluded, and nine randomized controlled trials enrolling 12,348 participants were included. No meta-analysis was performed. The original report-level exclusion log was unavailable; unsupported category counts were removed, and this reporting limitation is stated in the Results and Discussion.

The search retrieved 273 records. After 30 duplicates were removed, 243 records were screened by title and abstract and 216 were excluded. Twenty-seven full-text reports were assessed, of which nine randomized controlled trials met the criteria and 18 were excluded (Figure 2).

One trial included in the submitted version has been removed. Gao and colleagues (22) randomized participants between a series of Chinese herbal formulas adjusted daily according to traditional Chinese medicine syndrome and a single fixed herbal formula. Both arms received traditional Chinese medicine, so the trial has no placebo or standard-care comparator and does not meet the comparator criterion.

The original report-level exclusion log was unavailable. The comparator-based exclusion of Gao and colleagues could be verified, but the exact report-specific reasons for the other exclusions could not be reconstructed reliably. We therefore removed the unsupported category counts from the flow diagram and disclose this loss of screening documentation rather than assigning retrospective reasons without an auditable record. The available report-level information is summarized in Supplementary Table 1.

### Characteristics of included trials

The nine included trials were published between 2009 and 2025 and randomized 12,348 participants. Seven were conducted in mainland China, one in Singapore, and one was an international multicenter trial across Asia and Europe. Characteristics verified against primary publications, Supplementary material and registry records are in Table 2. The phase-stratified narrative synthesis is summarized in Table 3.

**Table 2:** Characteristics of included randomized controlled trials.

Trial	Reference	Registration	Design and centers	Clinical phase and window	Randomized/analyzed	Intervention	Comparator	Primary outcome	Published primary result	Safety as reported	Funding/source of support
Tongxinluo (TISS)	Dong et al. (8)	NCT01919671	Randomized, double-blind, placebo-controlled; 50 centers, China; Mar 2014 to Oct 2016	Acute. Within 72 h of onset. Age 35 to 75. NIHSS 4 to 22. First-ever stroke or prior stroke without disability	2,007 randomized; 1,979 safety; 1,946 mITT (973/973); 1,677 per protocol (840/837)	Tongxinluo capsules, 4 capsules orally three times daily for 90 days, plus guideline standard care	Matched placebo plus guideline standard care	Favorable functional outcome, mRS 0 to 1, at day 90	640/973 (65.8%) vs. 575/973 (59.1%); OR 1.33 (95% CI 1.11 to 1.60), *P* = 0.002; risk difference 6.68 (95% CI 2.38 to 10.95)	Any adverse event 382/989 (38.6%) vs. 380/990 (38.4%), *P* = 0.95. Serious adverse events 22/989 (2.2%) vs. 19/990 (1.9%), *P* = 0.64	Funded by Shijiazhuang Yiling Pharmaceutical Co. Ltd., which provided Tongxinluo and placebo capsules. The funder reportedly had no role in the study design, conduct, data analysis, manuscript preparation, or publication decision.
Panax notoginseng saponins (PANDA)	Wu et al. (9)	ChiCTR1800016363	Randomized, double-blind, placebo-controlled; 67 tertiary centers, China; Jul 2018 to Jun 2020	Acute. Within 14 days of onset. Age 18 to 75. NIHSS 4 to 15	3,072 randomized; 2,966 mITT (1,487/1,479); safety cohort 1,488/1,482	Xuesaitong soft capsules 120 mg orally twice daily for 3 months, plus standard care	Matched placebo 120 mg orally twice daily plus standard care	Functional independence, mRS 0 to 2, at 3 months	1,328/1,487 (89.3%) vs. 1,218/1,479 (82.4%); OR 1.95 (95% CI 1.56 to 2.44), *P* < 0.001	Safety cohort 1,488 vs. 1,482. Any adverse event 2.8% vs. 3.6%, *P* = 0.20. Serious adverse events 15/1,488 (1.0%) vs. 16/1,482 (1.1%), *P* = 0.85. Symptomatic intracranial hemorrhage 0.1% vs. 0%, *P* = 0.32. All-cause mortality 0.1% vs. 0.1%, *P* = 0.56	Funded by grant GN-2017B0003 from the Program of Research and Popularization of Appropriate Intervention Technology for the Stroke High Risk Group in China and China Resources Kunming Shenghuo Pharmaceutical Co. Ltd. The funders reportedly had no role in the study design, conduct, data analysis, manuscript preparation, or publication decision.
Angong Niuhuang Pills (ANGONG TRIAL)	Li et al. (23)	NCT04475328	Randomized, double-blind, placebo-controlled pilot; 17 centers, China; Apr 2021 to Jul 2022	Acute. Within 36 h of onset. Age 40 to 80. NIHSS 10 to 20. Anterior circulation infarction	120 enrolled, 60 per arm; 117 full analysis set (57/60); 96 per protocol (47/49)	Angong Niuhuang Pills 3 g/pill, 1 pill daily for 5 days, orally or nasally, plus guideline standard care	Matched placebo plus guideline standard care	Change in cerebral infarct and edema volume at day 14. Primary safety outcome: serious adverse events to day 90	Infarct volume change 0.3 vs. 0.4 ml; median difference −7.1 ml (IQR −18.3 to 2.3), *P* = 0.30. Edema volume change 11.4 vs. 4.0 ml; median difference 3.0 ml (IQR −1.3 to 9.9), *P* = 0.15. Both primary outcomes negative	Serious adverse events to day 90: 3/57 (5%) vs. 7/60 (12%), *P* = 0.36. Any adverse event to day 90: 14/57 (25%) vs. 12/60 (20%), *P* = 0.55. All-cause death 3 (5%) vs. 2 (3%), *P* = 0.95. Serum mercury and arsenic comparable	National Health Commission Stroke Prevention Project (GN-2020B0001) and Tongrentang Pharmaceutical Factory, Beijing Tongrentang Co.
NeuroAiD/MLC601 (CHIMES)	Chen et al. (14)	NCT00554723	Randomized, double-blind, placebo-controlled; international multicenter (Asia and Europe)	Acute. Within 72 h of onset. Age above 21. NIHSS 6 to 14	1,100 randomized; 1,006 with baseline and 3-month mRS data in the prognostic analysis	MLC601 (NeuroAiD) for 3 months, plus standard care	Matched placebo plus standard care	Shift in the modified Rankin Scale at 3 months	Adjusted OR 1.09 (95% CI 0.86 to 1.32). No statistical difference on any secondary outcome. Trial negative	Serious and non-serious adverse event rates similar between groups. Counts not given in the retrieved report	Not reported in the available primary publication.
Xueshuan Xinmai tablets	Wei et al. (24)	ChiCTR-TRC-12003074 (registered 9 Nov 2012, after enrollment began)	Randomized, double-blind, placebo-controlled; single center, First Affiliated Hospital of Anhui College of TCM; Jan to Dec 2012	Acute. Age 40 to 80. NIHSS 3 to 10. Time from onset not specified in the primary report	44 randomized (22/22); 42 analyzed after 2 withdrawals; 5 further excluded from imaging analysis for head motion	Xueshuan Xinmai tablets (TianXinTai) 0.8 g orally three times daily for 2 weeks, plus aspirin, atorvastatin and supportive care	Matched placebo tablets 0.8 g orally three times daily plus the same background therapy	Brain functional connectivity on resting-state fMRI, with NIHSS and SSQOL as clinical measures	Group by time interaction significant for NIHSS (F = 5.402, *P* = 0.026), SSQOL (F = 9.755, *P* = 0.003) and hematocrit (F = 4.601, *P* = 0.039). No effect on infarct volume. Between-group effect estimates with confidence intervals are not reported	Vital signs and adverse events recorded at each visit. No adverse event counts reported	Study drug and placebo supplied by Jilin Huakang Pharmaceutical Co.
Qizhitongluo capsule	Yu et al. (13)	NCT01762163	Randomized, double-blind, placebo- and active-controlled, three-arm adaptive design; 13 sites, China; Oct 2013 to Oct 2016	Recovery phase. Treatment started 15 to 28 days after onset. Age 35 to 80. NIHSS 4 to below 20. Fugl-Meyer below 90 or aphasia. Qi deficiency and blood stasis syndrome	622 randomized 2:1:1: Qizhitongluo 309, Naoxintong 159, placebo 154	Qizhitongluo capsule for 12 weeks, plus aspirin and routine recovery training	Two comparators: matched placebo and Naoxintong capsule (active), both plus aspirin and routine recovery training	Change in Lower Limb Fugl-Meyer Motor Scale score from baseline over 12 weeks	Qizhitongluo vs. placebo at week 12: difference 1.81 (95% CI 0.88 to 2.74), *P* = 0.0001. FMMS-LL increased 4.81 (QZTL), 3.77 (NXT) and 3.00 (placebo)	Adverse events, all-cause mortality and new cardiovascular events pre-specified as secondary outcomes over 20 weeks. Counts not given in the retrieved report	China Academy of Chinese Medical Sciences.
NeuroAiD/MLC601 (TIERS)	Kong et al. (15)	None reported in the primary publication. Registration status was not established from the available primary report.	Randomized, double-blind, placebo-controlled phase II pilot; rehabilitation setting, Singapore	Early recovery. Recent ischemic stroke, less than 1 month from onset	40 randomized. Exact per-arm randomized and analyzed numbers were not reported in the available source.	MLC601 (NeuroAiD), 4 capsules three times daily for 4 weeks, plus inpatient rehabilitation	Matched placebo plus inpatient rehabilitation	Fugl-Meyer Assessment, NIHSS and Functional Independence Measure at baseline, 4 and 8 weeks	No outcome reached statistical significance. Fugl-Meyer showed a positive trend favoring MLC601. Subgroups with posterior circulation infarction and severe stroke showed a tendency toward better recovery. Trial negative	Not reported in the retrieved report	Not reported in the available primary publication.
Naoxintong capsule	Yu et al. (11)	NCT02334969	Randomized, double-blind, placebo-controlled; 23 centers, China; Mar 2015 to Mar 2016	Secondary prevention. Adults with a history of ischemic stroke	2,200 enrolled; intention-to-treat analysis; 143 and 158 lost to follow-up	Naoxintong capsule 1.2 g orally twice daily, plus standard care	Matched placebo plus standard care	Recurrence of ischemic stroke within 2 years	6.5% vs. 9.5%; HR 0.665 (95% CI 0.492 to 0.899), *P* = 0.008. No significant difference in secondary outcomes	No significant difference in severe hemorrhage, cerebral hemorrhage or subarachnoid hemorrhage (all *P* > 0.05). Counts not given in the retrieved report	Not reported in the available primary publication.
Dengzhan Shengmai capsule	Cai et al. (12)	NCT00548223 (SPIRITDZSM1)	Randomized, double-blind, placebo-controlled; 83 hospitals, mainland China; Dec 2007 to Jan 2011	Secondary prevention. 14 to 180 days after first ischemic stroke. Age 40 to 75. At least one modifiable vascular risk factor	3,143 randomized; intention-to-treat analysis. Exact per-arm analyzed denominators were not established; published counts and percentages are reported without deriving denominators.	Dengzhan Shengmai capsule 0.36 g orally twice daily for 12 months, plus standard secondary prevention	Matched placebo plus standard secondary prevention	One-year incidence of stroke	58 (3.8%) vs. 82 (5.4%); HR 0.70 (95% CI 0.50 to 0.98), *P* = 0.036	Type and incidence of adverse events similar between groups. Counts not given in the retrieved report	Guangzhou University of Traditional Chinese Medicine.

**Table 3:** SWiM synthesis by clinical phase and formulation.

Clinical phase	Formulation (trial)	Primary outcome	Analysis population	Published effect estimate	Synthesis category
Acute treatment (five trials, 6,343 randomized)	Tongxinluo (TISS)	mRS 0 to 1 at day 90	mITT, 1,946	640/973 (65.8%) vs. 575/973 (59.1%); OR 1.33 (95% CI 1.11 to 1.60); risk difference 6.68 (95% CI 2.38 to 10.95)	Benefit on primary outcome
	Panax notoginseng saponins (PANDA)	mRS 0 to 2 at 3 months	mITT, 2,966	1,328/1,487 (89.3%) vs. 1,218/1,479 (82.4%); OR 1.95 (95% CI 1.56 to 2.44)	Benefit on primary outcome
	Angong Niuhuang Pills (ANGONG)	Change in cerebral infarct and edema volume at day 14	Full analysis set, 117	Infarct: median difference −7.1 ml (IQR −18.3 to 2.3), *P* = 0.30. Edema: median difference 3.0 ml (IQR −1.3 to 9.9), *P* = 0.15	No clear difference
	NeuroAiD/MLC601 (CHIMES)	Shift in mRS at 3 months	1,100 randomized	Adjusted OR 1.09 (95% CI 0.86 to 1.32)	No clear difference
	Xueshuan Xinmai	None designated; specified result taken as NIHSS at 2 weeks	42 analyzed	Group by time interaction: NIHSS F = 5.402, *P* = 0.026; SSQOL F = 9.755, *P* = 0.003. No between-group effect estimate reported	Too incompletely reported to classify
Early recovery and rehabilitation (two trials, 662 randomized)	Qizhitongluo	Change in Lower Limb Fugl-Meyer Motor Scale over 12 weeks	Qizhitongluo 309 vs. placebo 154	Mean difference at week 12: 1.81 points (95% CI 0.88 to 2.74), *P* = 0.0001	Benefit on primary outcome
	NeuroAiD/MLC601 (TIERS)	Fugl-Meyer Assessment, NIHSS and Functional Independence Measure at 4 and 8 weeks	40 randomized	No outcome statistically significant; positive trend on Fugl-Meyer	No clear difference
Secondary prevention (two trials, 5,343 randomized)	Naoxintong	Recurrent ischemic stroke within 2 years	ITT, 2,200 enrolled	6.5% vs. 9.5%; HR 0.665 (95% CI 0.492 to 0.899), *P* = 0.008	Benefit on primary outcome
	Dengzhan Shengmai	Stroke incidence at 1 year	ITT, 3,143 randomized	58 (3.8%) vs. 82 (5.4%); HR 0.70 (95% CI 0.50 to 0.98), *P* = 0.036	Benefit on primary outcome

Effect measures are as published by each trial and are not directly comparable. No pooled estimate is presented. Synthesis categories are defined in the Methods and are based on each trial's primary outcome only; secondary outcomes and subgroup analyses were not used to assign a category. Only the Qizhitongluo vs. placebo comparison of the three-arm Qizhitongluo trial contributes to the synthesis. CI, confidence interval; HR, hazard ratio; IQR, interquartile range; ITT, intention to treat; mITT, modified intention to treat; mRS, modified Rankin Scale; NIHSS, National Institutes of Health Stroke Scale; OR, odds ratio; SSQOL, Stroke-Specific Quality of Life Scale.

Five trials evaluated acute treatment and enrolled 6,343 participants: Tongxinluo within 72 h of onset (8), Panax notoginseng saponins within 14 days (9), Angong Niuhuang Pills within 36 h (23), NeuroAiD (MLC601) within 72 h in the CHIMES trial (14), and Xueshuan Xinmai tablets in the acute phase (24). Two trials evaluated early recovery and rehabilitation and enrolled 662 participants: Qizhitongluo, started 15 to 28 days after onset (13), and NeuroAiD in the TIERS pilot, within 1 month of onset in a rehabilitation setting (15). Two trials evaluated secondary prevention and enrolled 5,343 participants: Naoxintong in patients with previous ischemic stroke (11) and Dengzhan Shengmai, started 14 to 180 days after a first ischemic stroke (12).

The eligible age ranges do not correspond to the broad adult population stated in the eligibility criteria. Only Panax notoginseng saponins (18 to 75 years) and CHIMES (older than 21 years) approached an unrestricted adult population. The others applied both lower and upper limits: 35 to 75 years for Tongxinluo, 35 to 80 for Qizhitongluo, 40 to 80 for Angong Niuhuang Pills and Xueshuan Xinmai, and 40 to 75 for Dengzhan Shengmai. Every trial specifying an age range excluded the oldest patients and most excluded younger adults, which is reflected in the indirectness judgments in Table 4.

**Table 4:** Summary of findings by formulation, clinical phase and outcome.

Phase	Formulation	Outcome and follow-up	Studies (participants)	Effect (95% CI)	RoB	Incons.	Indirect.	Impreci.	Pub. bias	Certainty	Downgrade footnotes
Acute	Tongxinluo	Favorable functional outcome, mRS 0 to 1, at 90 days	1 (1,946)	OR 1.33 (1.11 to 1.60); 65.8% vs. 59.1%	Not serious	Not assessable	Serious (−1)	Not serious	Not assessable	MODERATE	Indirectness: conducted entirely in China; ages 35 to 75; patients receiving endovascular therapy and those unable to swallow excluded. Risk of bias not downgraded: judged low risk in all five RoB 2 domains, with protocol and statistical analysis plan publicly available
Acute	Panax notoginseng saponins	Functional independence, mRS 0 to 2, at 3 months	1 (2,966)	OR 1.95 (1.56 to 2.44); 89.3% vs. 82.4%	Not serious	Not assessable	Serious (−1)	Not serious	Not assessable	MODERATE	Risk of bias was not downgraded because the trial used an automated centralized randomization system, double-blinding, and a pre-specified primary outcome that was consistent with the publicly available protocol and statistical analysis plan. Certainty was downgraded once for indirectness because the trial was conducted entirely in China, included participants aged 18 to 75 years, and had limited representation of patients receiving contemporary reperfusion treatment.
Acute	Angong Niuhuang Pills	Cerebral infarct and edema volume at 14 days	1 (117)	Infarct median difference −7.1 ml, *P* = 0.30	Serious (−1)	Not assessable	Serious (−1)	Serious (−1)	Not assessable	VERY LOW	Risk of bias: overall high risk from complete-case primary analysis. Indirectness: imaging surrogate rather than clinical outcome. Imprecision: pilot trial, no prior power calculation, wide intervals
Acute	NeuroAiD (MLC601)	Functional outcome, mRS shift, at 3 months	1 (1,100)	Adjusted OR 1.09 (0.86 to 1.32)	Serious (−1)	Not assessable	Not serious	Serious (−1)	Not assessable	LOW	Risk of bias: approximately 8.5% of randomized patients lacked the 3-month outcome and the handling of missing data could not be confirmed. Imprecision: no minimally important difference for an ordinal modified Rankin Scale shift was pre-specified in the trial or in this review, and the confidence interval (0.86 to 1.32) includes both no effect and effects that would be clinically important, so the outcome is downgraded once for imprecision.
Acute	Xueshuan Xinmai	Neurological deficit, NIHSS, at 2 weeks	1 (42)	No between-group estimate reported	Very serious (−2)	Not assessable	Not serious	Serious (−1)	Not assessable	VERY LOW	Risk of bias: overall high risk; registration post-dated enrollment and no between-group estimate was reported. Imprecision: 42 participants analyzed
Early recovery	Qizhitongluo	Lower-limb motor function, FMMS-LL, at 12 weeks	1 (463)	Mean difference 1.81 points (0.88 to 2.74)	Serious (−1)	Not assessable	Serious (−1)	Not serious	Not assessable	LOW	Risk of bias: adaptive design with planned interim analysis whose adaptation rules are not reported; completeness of primary outcome data not established. Indirectness: single country; enrollment restricted to one TCM syndrome pattern; no minimal clinically important difference defined
Early recovery	NeuroAiD (MLC601)	Motor recovery, Fugl-Meyer, at 4 and 8 weeks	1 (40)	No outcome statistically significant	Serious (−1)	Not assessable	Not serious	Very serious (−2)	Not assessable	VERY LOW	Risk of bias: randomization and attrition not described; no registration identified. Imprecision: 40 participants in a pilot not powered for efficacy
Secondary prevention	Naoxintong	Recurrent ischemic stroke at 2 years	1 (2,200)	HR 0.665 (0.492 to 0.899); 6.5% vs. 9.5%	Serious (−1)	Not assessable	Serious (−1)	Not serious	Not assessable	LOW	Risk of bias: 13.7% lost to follow-up for a time-to-event outcome; protocol not available. Indirectness: conducted entirely in China
Secondary prevention	Dengzhan Shengmai	Stroke incidence at 1 year	1 (3,143)	HR 0.70 (0.50 to 0.98); 3.8% vs. 5.4%	Serious (−1)	Not assessable	Serious (−1)	Not serious	Not assessable	LOW	Risk of bias: per-arm analyzed denominators cannot be reconciled with the randomized total; approximately 8 years between trial completion and publication. Indirectness: conducted entirely in China; ages 40 to 75. Imprecision not downgraded, but the upper confidence limit of 0.98 makes the result fragile

Certainty was rated using GRADE, starting at high for randomized evidence. Where a single trial informs an outcome, inconsistency cannot be assessed, and this is recorded as not assessable rather than as absence of concern. Publication bias could not be assessed formally for any outcome, because each formulation-outcome comparison was generally represented by a single trial. Qualitative concerns are described in the Discussion, but no outcome was downgraded for publication bias, because the available evidence was insufficient to support a defensible outcome-specific judgment. Risk-of-bias downgrades correspond to the overall RoB 2 judgments in Table 1 and Figure 1. No certainty rating is offered for traditional Chinese medicine as a treatment class, because no class-level intervention, outcome or effect was synthesized. CI, confidence interval; FMMS-LL, Lower Limb Fugl-Meyer Motor Scale; HR, hazard ratio; mRS, modified Rankin Scale; NIHSS, National Institutes of Health Stroke Scale; OR, odds ratio; RoB, risk of bias.

Analysis populations differed within trials as well as between them and are distinguished throughout. The Tongxinluo trial randomized 2,007 patients, included 1,979 in the safety analysis, 1,946 in the modified intention-to-treat analysis and 1,677 per protocol (8). The PANDA trial randomized 3,072, included 2,966 in the modified intention-to-treat cohort, and reported a safety cohort of 1,488 and 1,482 (9). The ANGONG trial enrolled 120, of whom 117 formed the full analysis set and 96 the per-protocol set (23). Efficacy results use efficacy denominators and safety results use safety denominators.

Two trials evaluated the same formulation: CHIMES and TIERS both tested MLC601, in the acute phase and in early recovery, respectively (14, 15). One formulation appears in two roles: Naoxintong is the subject of an included secondary-prevention trial (11) and is also an active comparator arm in the three-arm Qizhitongluo trial (13). Only the Qizhitongluo vs. placebo comparison contributes to this synthesis; the Naoxintong arm of that trial is not treated as independent evidence about Naoxintong.

Six trials reported funding or manufacturer involvement. The Tongxinluo trial was funded by Shijiazhuang Yiling Pharmaceutical Co Ltd, which supplied Tongxinluo and placebo, with the funder reported as having no role in study design, conduct, analysis, manuscript preparation or the decision to publish (8). The PANDA trial was funded by grant GN-2017B0003 from the Program of Research and Popularization of Appropriate Intervention Technology for the Stroke High Risk Group in China and by China Resources Kunming Shenghuo Pharmaceutical Co Ltd, with the funders reported as having no role in study design, conduct, analysis, manuscript preparation or the decision to publish (9). The ANGONG trial was supported in part by Tongrentang Pharmaceutical Factory, Beijing Tongrentang Co (23), and study medication for the Xueshuan Xinmai trial was supplied by Jilin Huakang Pharmaceutical Co (24). The Qizhitongluo trial was supported by the China Academy of Chinese Medical Sciences (13) and the Dengzhan Shengmai trial by Guangzhou University of Traditional Chinese Medicine (12).

**Figure 3:**
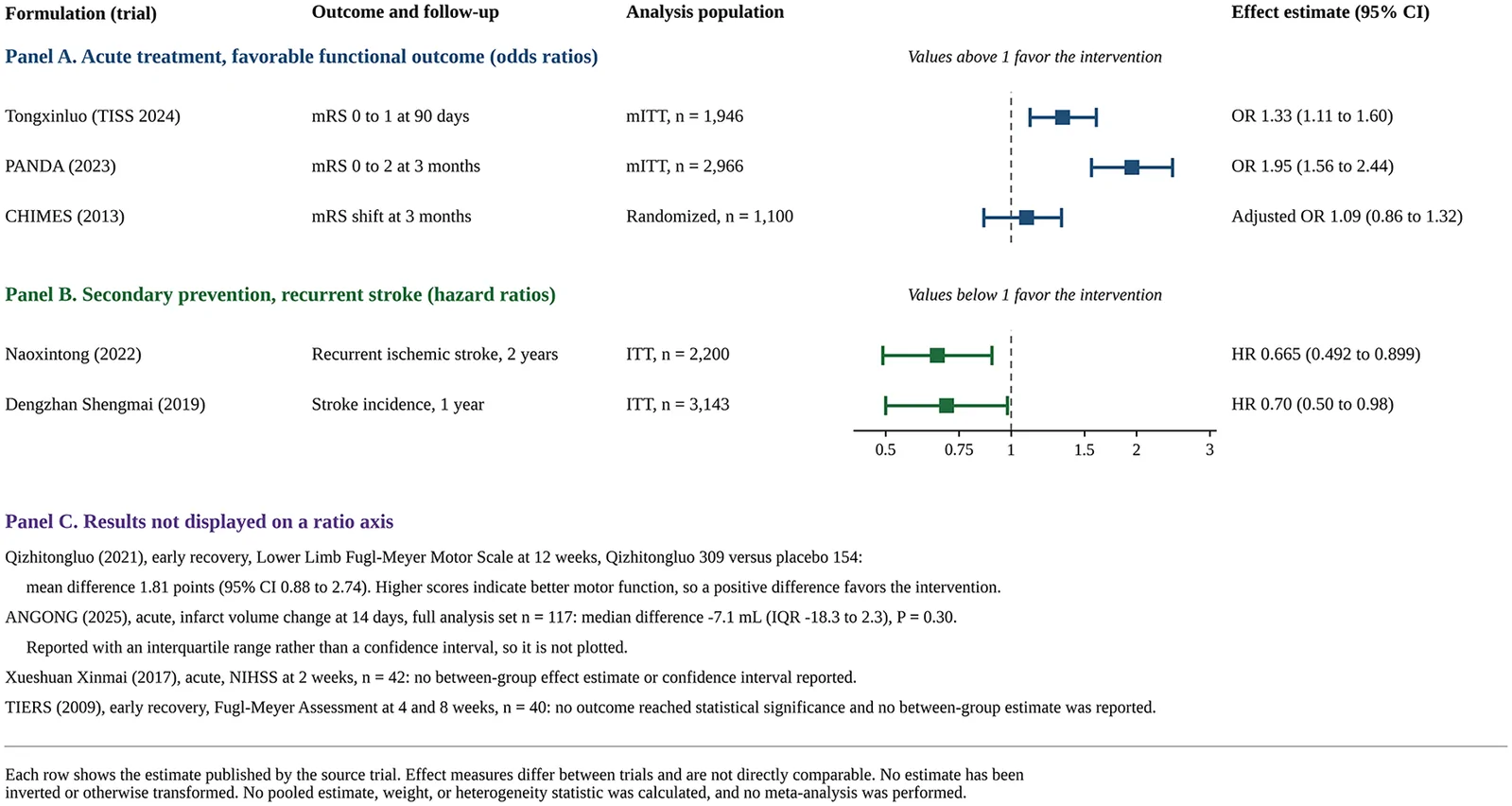
Trial-specific effect estimates for the primary outcome of each included trial, grouped by clinical phase. Each trial is shown separately with its formulation, outcome definition, follow-up interval, analysis population, published effect measure and 95% confidence interval. No summary estimate, pooled effect, weight or heterogeneity statistic is presented. Effect measures differ between trials and are not directly comparable; each is the measure reported by the source trial. Ratio measures are plotted on a logarithmic axis. The Qizhitongluo result is a mean difference on a continuous scale and is reported numerically rather than plotted on the ratio axis. Three trials that reported no between-group effect estimate with a confidence interval are listed with their results described in text.

Funding or manufacturer involvement is reported only where it could be established from the source material reviewed. Absence of a funding statement in Table 2 means that funding was not established- from the available report and should not be interpreted as absence of commercial support. Trial-specific primary-outcome estimates are displayed without pooling in Figure 3.

### Acute treatment

Two acute-phase trials reported benefit on their primary outcome. In the Tongxinluo trial, at 50 centers in China, 640 of 973 patients (65.8%) receiving Tongxinluo and 575 of 973 (59.1%) receiving placebo achieved mRS 0 to 1 at day 90 in the modified intention-to-treat analysis (OR 1.33, 95% CI 1.11 to 1.60; *P* = 0.002), with a reported absolute risk difference of 6.68 percentage points (95% CI 2.38 to 10.95) (8). In the PANDA trial, at 67 tertiary centers in China, 1,328 of 1,487 patients (89.3%) receiving Panax notoginseng saponins and 1,218 of 1,479 (82.4%) receiving placebo achieved functional independence, defined as mRS 0 to 2, at 3 months (OR 1.95, 95% CI 1.56 to 2.44; *P* < 0.001) (9). The absolute difference between arms in PANDA is 7.0 percentage points, a value calculated for this review from the published event counts rather than reported in the source publication.

These trials used related but non-identical definitions of a favorable outcome. An mRS score of 0 to 1 denotes no significant disability, whereas 0 to 2 denotes functional independence, a broader category. They are not interchangeable and the two results are not presented as estimates of the same quantity. The Tongxinluo trial also reported mRS 0 to 2 at 90 days as a secondary outcome, in 835 of 973 (85.8%) vs. 801 of 973 (82.3%) patients (OR 1.30, 95% CI 1.02 to 1.66), which is noted for completeness and is not combined with the PANDA result (8).

Two acute-phase trials showed no clear difference. The ANGONG pilot, at 17 centers, found a change in cerebral infarct volume at day 14 of 0.3 ml with Angong Niuhuang Pills and 0.4 ml with placebo (median difference −7.1 ml, interquartile range −18.3 to 2.3; *P* = 0.30), and a change in cerebral edema volume of 11.4 and 4.0 ml, respectively (median difference 3.0 ml, interquartile range −1.3 to 9.9; *P* = 0.15). Both primary outcomes were negative. A sensitivity analysis restricted to patients with large-artery atherosclerosis found a reduction in infarct volume (median difference −12.3 ml, interquartile range −27.7 to −0.3; *P* = 0.03), which is a subgroup finding within a negative pilot and is hypothesis-generating only (23). The CHIMES trial randomized 1,100 patients within 72 h of onset and found an adjusted odds ratio of 1.09 (95% CI 0.86 to 1.32) for the shift in mRS at 3 months, with no statistical difference on any secondary outcome (14).

One acute-phase trial was too incompletely reported to classify. A single-center trial randomized 44 patients to Xueshuan Xinmai tablets 0.8 g three times daily or matched placebo for 2 weeks, with aspirin, atorvastatin and supportive care in both arms. The report does not designate a primary outcome; it presents resting-state functional connectivity on magnetic resonance imaging alongside NIHSS and Stroke-Specific Quality of Life Scale scores. Improvement was reported in the treatment arm as group by time interaction terms from repeated-measures analysis of variance (NIHSS F = 5.402, *P* = 0.026; SSQOL F = 9.755, *P* = 0.003), with no between-group effect estimate or confidence interval for any clinical outcome, and the interval between stroke onset and enrollment was not specified (24). For risk-of-bias assessment the specified result was taken to be the change in NIHSS at 2 weeks, selected as the most clinically relevant reported outcome and identified as such because the report designates none.

### Early recovery and rehabilitation

The Qizhitongluo trial randomized 622 patients at 13 sites in a 2:1:1 ratio to Qizhitongluo, Naoxintong or placebo, starting 15 to 28 days after onset and continuing 12 weeks, with aspirin and routine recovery training in all arms. In the comparison contributing to this review, Qizhitongluo (*n* = 309) vs. placebo (*n* = 154), the Lower Limb Fugl-Meyer Motor Scale score increased by 4.81 and 3.00 points respectively, a difference at week 12 of 1.81 points (95% CI 0.88 to 2.74; *P* = 0.0001), meeting the definition of benefit on the primary outcome (13). Whether a difference of this size on this scale is clinically important is uncertain, and the trial did not specify a minimal clinically important difference.

The TIERS trial was a phase II pilot randomizing 40 patients with ischemic stroke of less than 1 month duration to MLC601 or placebo for 4 weeks in a rehabilitation setting. No outcome reached statistical significance. Fugl-Meyer Assessment scores showed a positive trend favoring MLC601, and subgroups with posterior circulation infarction and with severe stroke showed a tendency toward better recovery, but the trial was designed to inform larger studies rather than to establish efficacy (15).

The two trials of MLC601 in this review, one acute and one in early recovery, both failed to demonstrate benefit on their primary outcomes.

### Secondary prevention

Both secondary-prevention trials reported benefit. The Naoxintong trial enrolled 2,200 adults with a history of ischemic stroke at 23 centers, comparing Naoxintong capsule 1.2 g twice daily with placebo in addition to standard care. Recurrence of ischemic stroke within 2 years occurred in 6.5 and 9.5%, respectively (HR 0.665, 95% CI 0.492 to 0.899; *P* = 0.008). There were no significant differences in myocardial infarction, death from recurrent stroke or all-cause mortality, and none in severe hemorrhage, cerebral hemorrhage or subarachnoid hemorrhage. In total 301 participants, 143 and 158 by arm, were lost to follow-up (11).

The Dengzhan Shengmai trial randomized 3,143 patients at 83 hospitals, 14 to 180 days after a first ischemic stroke, to Dengzhan Shengmai capsule 0.36 g twice daily or placebo for 12 months in addition to standard secondary prevention. New stroke events occurred in 58 participants (3.8%) and 82 (5.4%), respectively (HR 0.70, 95% CI 0.50 to 0.98; *P* = 0.036), with similar type and incidence of adverse events (12). The upper limit of the confidence interval approaches unity and the result is correspondingly fragile.

The report provided event counts and percentages but the per-arm analyzed denominators could not be reconciled confidently with the randomized total from the material available for this revision. We therefore report the published counts, percentages and hazard ratio without deriving denominators from rounded percentages.

### Risk of bias

Risk of bias for the specified result in each trial is shown in Figure 1 and Table 1, with domain-level rationales and supporting source text in Supplementary Table 2. Judgments varied across trials. The Tongxinluo trial was judged at low risk of bias in all five domains and overall: randomization numbers were generated by an independent statistician using a centralized automated system, allocation was handled independently, placebo was matched for color, odor, taste, shape, texture, specification, appearance, packaging and labeling, 96.5% of randomized patients contributed to the primary analysis, and the study protocol and statistical analysis plan are publicly available with the published report, allowing the reported primary outcome to be checked against the planned one. The PANDA trial was likewise judged at low risk of bias in all five domains and overall: randomization codes were generated by an automated centralized randomization system, the trial was multicenter, double-blind and placebo-controlled, and the trial protocol and statistical analysis plan are publicly available with the published report, so the reported primary outcome could be checked against the planned one.

Five trials were judged to raise some concerns, most often because sequence generation or allocation concealment was not described, because the completeness of primary outcome data could not be established, or because no protocol was available against which to check the reported result. Two trials were judged at high risk: the ANGONG trial, because its primary efficacy analysis was a complete-case analysis with approximately one in six participants lacking primary outcome data; and the Xueshuan Xinmai trial, because registration post-dated the start of enrollment and no between-group effect estimate was reported for any clinical outcome.

Figure 1 and Table 1 in the submitted version contradicted one another, the figure showing high risk for selection of the reported result in all ten trials while the table recorded some concerns, and the two disagreeing on the first four domains for the two largest trials. Both are now generated from a single master dataset. Overall judgments follow the RoB 2 algorithm and are never more favorable than the least favorable contributing domain.

### Certainty of evidence

Certainty was rated separately for each formulation, clinical phase, and outcome. The GRADE evidence profiles are presented in Table 4. No outcome was rated high certainty. Two were rated moderate: the favorable functional outcome with Tongxinluo, downgraded once for indirectness but not for risk of bias, since that trial was judged at low risk of bias in every domain; and functional independence with Panax notoginseng saponins in PANDA, also downgraded once for indirectness but not for risk of bias, after the trial was reassessed against its publicly available protocol and statistical analysis plan. Four outcomes were rated low certainty and three very low. The rating for the null result of MLC601 at 3 months in CHIMES was revised during this revision. The submitted version treated the confidence interval around the adjusted odds ratio of 1.09 (0.86 to 1.32) as narrow enough to exclude a clinically important benefit, but no minimally important difference for an ordinal modified Rankin Scale shift was pre-specified in that trial or in this review, so the claim could not be defended. Because the interval includes both no effect and effects that many readers would consider clinically important, the outcome is now downgraded once for imprecision in addition to once for risk of bias and is rated low certainty. Ratings were derived from the corrected risk-of-bias judgments rather than assigned independently of them.

We have not rated certainty for traditional Chinese medicine considered as a treatment class, because no class-level intervention, outcome or effect was synthesized and a Summary of Findings row without an underlying synthesis would be meaningless. The relevant point is made in the Discussion instead: the trials evaluate eight formulations with different compositions across three clinical phases, results differ in direction between formulations, and no class-level conclusion can be drawn from them at any level of certainty.

### Safety

Trial-level safety data are in Table 5. Safety reporting was incomplete across the evidence base, and the review cannot establish the comparative safety of adjunctive traditional Chinese medicine.

**Table 5:** Trial-level safety data.

Trial	Safety population	Follow-up	Any adverse event	Serious adverse events	Intracranial hemorrhage	Mortality	Discontinuation	Herb-drug interaction
Tongxinluo	989 vs. 990	90 days	382 (38.6%) vs. 380 (38.4%), *P* = 0.95	22 (2.2%) vs. 19 (1.9%), *P* = 0.64	Hemorrhagic stroke 4 (0.4%) vs. 3 (0.3%)	Vascular death 2 (0.2%) vs. 6 (0.6%)	Not reported	Not reported
PANDA	1,488 vs. 1,482	3 months	2.8% vs. 3.6%, *P* = 0.20	15 (1.0%) vs. 16 (1.1%), *P* = 0.85	Symptomatic intracranial hemorrhage 0.1% vs. 0%, *P* = 0.32	All-cause mortality 0.1% vs. 0.1%, *P* = 0.56	Not reported	Not reported
ANGONG	57 vs. 60	90 days	14 (25%) vs. 12 (20%), *P* = 0.55	3 (5%) vs. 7 (12%), *P* = 0.36	Not reported separately	All-cause death 3 (5%) vs. 2 (3%), *P* = 0.95	Not reported	Serum mercury and arsenic comparable at day 7
CHIMES	Not reported	3 months	Described as similar between arms; counts not reported	Described as similar between arms; counts not reported	Not reported	Not reported	Not reported	Not reported
Xueshuan Xinmai	Not reported	2 weeks	Recorded at each visit; counts not reported	Not reported	Not reported	Not reported	Not reported	Not reported
Qizhitongluo	Not reported	20 weeks	Pre-specified secondary outcome; counts not reported	Not reported	Not reported	All-cause mortality pre-specified; counts not reported	Not reported	Not reported
TIERS	Not reported	8 weeks	Not reported	Not reported	Not reported	Not reported	Not reported	Not reported
Naoxintong	Not reported	2 years	Not reported	Not reported	No significant difference in severe hemorrhage, cerebral hemorrhage or subarachnoid hemorrhage; counts not reported	All-cause mortality: no significant difference; counts not reported	Not reported	Not reported
Dengzhan Shengmai	Not reported	12 months	Type and incidence similar between arms; counts not reported	Not reported	Not reported	Not reported	Not reported	Not reported

Entries recorded as not reported indicate that the outcome was not reported in the primary publication. Not reported is not the same as zero events and must not be interpreted as such. Safety reporting was incomplete across the evidence base, and this review cannot establish the comparative safety of adjunctive traditional Chinese medicine. Absence of reported harm is not evidence of absence of harm. Six of the nine trials, including four large trials, did not report adverse events by arm.

Three trials reported adverse-event counts by arm. In the Tongxinluo trial, in a safety population of 989 and 990 patients, adverse events occurred in 382 (38.6%) and 380 (38.4%), respectively (*P* = 0.95) and serious adverse events in 22 (2.2%) and 19 (1.9%; *P* = 0.64); hemorrhagic stroke occurred in 4 (0.4%) and 3 (0.3%) patients and vascular death in 2 (0.2%) and 6 (0.6%) (8). In the PANDA trial, in a safety cohort of 1,488 and 1,482 patients, any adverse event occurred in 2.8 and 3.6%, respectively (*P* = 0.20) and serious adverse events in 15 (1.0%) and 16 (1.1%; *P* = 0.85); symptomatic intracranial hemorrhage occurred in 0.1% and 0% (*P* = 0.32) and all-cause mortality was 0.1% in both arms (*P* = 0.56) (9). In the ANGONG trial, serious adverse events within 90 days occurred in 3 of 57 (5%) and 7 of 60 (12%; *P* = 0.36), adverse events in 14 (25%) and 12 (20%), and all-cause death in 3 (5%) and 2 (3%); serum mercury and arsenic concentrations at day 7 were comparable between arms, which matters because Angong Niuhuang Pills contain realgar and cinnabar (23).

The criterion applied throughout is whether the primary publication reported adverse events separately for each randomized arm, as counts or as arm-specific percentages. Three trials met it, and the remaining six described adverse events qualitatively as similar between arms or did not report them at all. Four of these are large trials, and the absence of arm-level counts in their published reports is itself a finding about this literature. Entries recorded as not reported in Table 5 indicate that the outcome was not reported and must not be read as zero events. No trial systematically assessed herb-drug interactions or interactions with antithrombotic therapy. Absence of reported harm is not evidence of absence of harm, and the data assembled here do not support any statement that adjunctive traditional Chinese medicine is safe in this population.

For trials without verifiable arm-level adverse-event counts or a clearly defined safety population in the available reports, Table 5 records the item as not reported. No missing value was treated as zero, and comparative safety is not inferred from incomplete reporting.

## Discussion

This review identified nine randomized controlled trials of adjunctive traditional Chinese medicine in ischemic stroke, enrolling 12,348 participants across three clinical phases and eight formulations. The trials do not address a single clinical question, and the evidence does not support a conclusion about traditional Chinese medicine as a treatment class.

Five trials reported benefit on their primary outcome, three did not, and one was too incompletely reported to classify. That pattern is not a vote count and should not be read as one. The two largest acute-phase trials each reported a modest improvement in functional outcome at 3 months, with absolute differences of approximately 7 percentage points. The Tongxinluo and PANDA results each carry moderate certainty, the highest rating for any benefit in this review, reflecting trials judged at low risk of bias for the assessed result with publicly available protocols and statistical analysis plans; both are nonetheless downgraded for indirectness, having been conducted entirely in China, in patients aged 35 to 75 years and 18 to 75 years respectively, with limited representation of patients receiving contemporary reperfusion treatment. Neither formulation has been replicated outside China.

The two secondary-prevention trials are the largest in the review and consistent in direction, each reporting an approximately 30% relative reduction in recurrent stroke, yet both were rated low certainty. The Dengzhan Shengmai result is statistically fragile, with an upper confidence limit of 0.98, and the Naoxintong trial lost 13.7% of participants to follow-up. Neither has been replicated. These two trials address the clinical question with the clearest patient-relevant endpoint and the longest follow-up, and they received the least attention in the submitted version of this review, where they were described as providing insufficient extractable data.

MLC601 has now been evaluated in two randomized trials here, in the acute phase and in early recovery, and neither demonstrated benefit on its primary outcome. CHIMES was the larger of the two and its null result is the more informative, although it carries low certainty because the confidence interval around the adjusted odds ratio is compatible with both no effect and a clinically important benefit and no minimally important difference was pre-specified. This is a useful counterweight to any impression that randomized evidence in this field is uniformly favorable, and it is the clearest illustration of why interpretation must be formulation-specific. Results differ in direction between formulations tested in comparable settings, which is precisely why a class-level certainty rating would be uninformative and why none is offered.

### Formulation differences

The formulations evaluated here are not variants of one another. Tongxinluo is a compound preparation of twelve herbal and animal-derived components, developed originally for cardiovascular indications, with reported vasodilatory, antiplatelet, anticoagulant, thrombolytic and lipid-lowering properties. Panax notoginseng saponins, marketed as Xuesaitong, is a purified saponin extract of a single plant and is closer in character to a defined pharmacological agent than to a compound prescription. NeuroAiD (MLC601) is a multi-component herbal and animal-derived preparation developed for post-stroke recovery. Angong Niuhuang Pills is a classical formulation whose constituents include realgar and cinnabar, arsenic-containing and mercury-containing minerals respectively, raising heavy-metal exposure questions that do not arise with the other preparations (23, 25, 26). Naoxintong, Dengzhan Shengmai, Xueshuan Xinmai and Qizhitongluo are compound patent medicines of differing composition, several formulated around the principle of invigorating qi and activating blood circulation.

These differences imply different plausible mechanisms, different safety profiles, different potential for interaction with antithrombotic therapy, and different manufacturing and quality-control requirements. They are described here to explain why the evidence must be read formulation by formulation. They are not offered as support for any clinical conclusion. Mechanistic plausibility is not evidence of clinical effect, and nothing in this section strengthens any efficacy claim made in the Results.

### Strengths and limitations

The review searched international and Chinese-language sources, used PRISMA 2020 and PRISMA-S as reporting frameworks and applied SWiM, RoB 2 and GRADE, verified the characteristics of every included trial against its primary report and registry record, and separated evidence by clinical phase and formulation rather than aggregating it. Analysis populations are distinguished throughout and effect measures are reported as the source trials published them.

The limitations are substantial. The review was not prospectively registered and no dated protocol preceded screening, so it cannot be verified that eligibility criteria, outcomes and analytic decisions were fixed before the results were known. Documentation of the review process is incomplete: the original record of reasons for excluding full-text reports was incomplete, and the executed search strategies and the record of which decisions were made independently and in duplicate were not available. These gaps reduce confidence in the reproducibility of the review process itself, independently of the quality of the included trials, and they are the reason this review should be read as a structured description of the available randomized evidence rather than as a fully reproducible synthesis. Consequently, the affected PRISMA 2020 and PRISMA-S reporting items remain incomplete.

The evidence base is geographically concentrated, with seven of nine trials conducted entirely in mainland China and an eighth in Singapore. Every trial specifying an age range excluded older patients. Most trials excluded patients receiving endovascular therapy or were conducted before it became widely available, so the evidence speaks poorly to contemporary practice where reperfusion therapy is routine. Every outcome is supported by a single trial, so consistency could not be assessed and no finding has been independently replicated.

Publication bias could not be assessed formally, because each formulation-outcome comparison was generally represented by a single trial. Qualitative concerns remain and are not negligible. Trials of traditional Chinese medicine conducted in China have historically shown a high proportion of positive results, four included trials reported pharmaceutical industry funding or manufacturer-supplied study medication, among them both large acute-phase trials that reported benefit, and small negative trials in this field may be less likely to reach international indexing. Commercial funding does not by itself demonstrate that bias has occurred; it is a reason for careful interpretation and for transparent reporting of the sponsor role, which the two largest trials provide. These are reasons to interpret the positive findings cautiously rather than a demonstration that publication bias has occurred. No outcome was downgraded for publication bias, because the available evidence was insufficient to support a defensible outcome-specific judgment.

Incomplete reporting in the included trials constrains what this review can say. A quantitative sensitivity analysis excluding poorly reported trials was not applicable, because the synthesis contains no pooled estimate that such an exclusion could change. The equivalent step in a narrative synthesis is to identify which conclusions rest on trials with reporting deficiencies. Three do: the classification of the Xueshuan Xinmai trial, which reports no between-group effect estimate; the interpretation of the large-artery atherosclerosis subgroup finding in the ANGONG trial, which arises within a negative pilot; and the safety conclusions for six of the nine trials. Each is correspondingly weak.

### Implications for practice and research

This review does not support the routine use of adjunctive traditional Chinese medicine in ischemic stroke, and nothing in it should be read as a recommendation for clinical adoption. Individual formulations produced trial-specific findings that require independent confirmation before they could inform practice. Certainty is moderate for two positive results and low or very low for the rest, no result has been replicated, and comparative safety has not been established.

Future trials should be prospectively registered with publicly accessible protocols and statistical analysis plans, adequately powered for patient-relevant endpoints, and conducted with concealed allocation and blinded outcome assessment. Functional endpoints should be harmonized so results can be compared across trials, and the analysis population for every reported result should be stated. Trials should report formulation composition, dose, route, manufacturing standard and batch quality in enough detail to permit replication.

Safety reporting requires particular improvement. Future trials should use pre-defined adverse-event definitions, active rather than passive surveillance, formal seriousness grading, causality assessment and complete reporting of treatment discontinuations, with follow-up long enough to detect delayed events. Two requirements are specific to this field: interactions with antiplatelet and anticoagulant therapy should be assessed systematically and prospectively rather than noted incidentally, given that several of these preparations are described as having antiplatelet or anticoagulant properties; and biological monitoring should be routine for preparations containing heavy metals, as in the ANGONG trial, which measured serum mercury and arsenic. Replication outside China, and in populations with access to contemporary reperfusion therapy, is needed before any of these formulations could be considered for wider use.

## Conclusion

Nine randomized trials have evaluated eight traditional Chinese medicine formulations as adjuncts to standard care in ischemic stroke, across acute treatment, early recovery and secondary prevention. Five reported benefit on their primary outcome, three did not, and one was too incompletely reported to classify. Certainty was moderate for two positive results and low or very low for all others; no finding has been independently replicated, and safety reporting was too incomplete to establish comparative safety. The evidence does not support conclusions about traditional Chinese medicine as a class of treatment and does not support the routine clinical use of any individual formulation. Prospectively registered, adequately powered, independently replicated trials with harmonized endpoints and standardized safety reporting are required.

## Data Availability

All data underlying this review derive from previously published studies cited in the references and are summarized in the manuscript and its supplementary material. The search-source record is provided in Supplementary Appendix 1, the available report-level full-text exclusion information in Supplementary Table 1, detailed RoB 2 judgments and supporting rationales in Supplementary Table 2, and the extracted data underlying Figures 1 and 3 and Tables 1 to 5 in Supplementary Data File 1. Together, these materials provide an audit trail for the PRISMA counts, trial-specific effect estimates, RoB 2 judgments, GRADE profiles, and safety summaries. They contain extracted aggregate data from published reports and no unpublished patient-level data.
