# Supplementary material for: Adjunctive traditional Chinese medicine for ischemic stroke: a systematic review with structured narrative synthesis and GRADE assessment of randomized controlled trials
Source: Front Med (Lausanne). 2026 Sep 4;13:1884399. doi: 10.3389/fmed.2026.1884399 (PMC13585579; doi:10.3389/fmed.2026.1884399)
Supplement: Supplementary file 1 [file Supplementary_File_1.docx]

**Supplementary Appendix 1. Search information retained and unavailable**

The original database-specific search histories and exact executed strings were not retained in the files available for this revision. This appendix is therefore a record of what was retained and what was not. It is not a set of reproducible search strategies, and no retrospectively reconstructed syntax is presented as the search originally run. The table lists the sources that were searched, together with the status of each item of execution detail. The main manuscript identifies this loss of documentation as a limitation of the review.

| **Source** | **Type** | **Platform or interface** | **Status of execution date** | **Status of records retrieved** | **Status of the executed search string** |
| --- | --- | --- | --- | --- | --- |
| PubMed | Bibliographic database | Not retained | Search completed by 8 April 2026; source-specific date not retained | Source-specific count not retained | Exact executed string not retained |
| Embase | Bibliographic database | Not retained | Search completed by 8 April 2026; source-specific date not retained | Source-specific count not retained | Exact executed string not retained |
| Web of Science | Bibliographic database | Not retained | Search completed by 8 April 2026; source-specific date not retained | Source-specific count not retained | Exact executed string not retained |
| Cochrane CENTRAL | Bibliographic database | Not retained | Search completed by 8 April 2026; source-specific date not retained | Source-specific count not retained | Exact executed string not retained |
| CNKI | Bibliographic database | Not retained | Search completed by 8 April 2026; source-specific date not retained | Source-specific count not retained | Exact executed string not retained |
| Wanfang Data | Bibliographic database | Not retained | Search completed by 8 April 2026; source-specific date not retained | Source-specific count not retained | Exact executed string not retained |
| SinoMed | Bibliographic database | Not retained | Search completed by 8 April 2026; source-specific date not retained | Source-specific count not retained | Exact executed string not retained |
| Chinese Clinical Trial Registry | Clinical trial registry | Not retained | Search completed by 8 April 2026; source-specific date not retained | Source-specific count not retained | Exact executed string not retained |

Note. MEDLINE content was searched through the PubMed interface and was not counted as a separate database. The final source count is seven bibliographic databases and one clinical trial registry.
