# Supplementary material for: Adjunctive traditional Chinese medicine for ischemic stroke: a systematic review with structured narrative synthesis and GRADE assessment of randomized controlled trials
Source: Front Med (Lausanne). 2026 Sep 4;13:1884399. doi: 10.3389/fmed.2026.1884399 (PMC13585579; doi:10.3389/fmed.2026.1884399)
Supplement: Supplementary file 2 [file Table_1.docx]

Supplementary Table 1. Full-text reports excluded, with available reasons

The original report-level exclusion log was unavailable. The exclusion of Gao and colleagues was verified from the published comparator, but report-specific reasons for the remaining exclusions could not be reconstructed reliably and have not been assigned retrospectively. Unsupported category counts were removed from the PRISMA diagram, and the loss of screening documentation is disclosed in the manuscript.

| **No.** | **Full citation** | **Primary reason for exclusion** |
| --- | --- | --- |
| 1 | Gao Y, Zhong H, Ma B, Huang Y. Randomized, double-blind, parallel-controlled clinical trial on correspondence of prescriptions and traditional Chinese medicine syndrome of ischemic stroke. J Tradit Chin Med. 2012;32(3):335-342. doi:10.1016/s0254-6272(13)60034-5 | Ineligible comparator. Participants were randomized between a series of Chinese herbal formulas adjusted daily by syndrome and a single fixed herbal formula. Both arms received traditional Chinese medicine, so there was no placebo or standard-care-only comparator. This trial is reference 22 in the main manuscript and is cited there only as an excluded report. |
| 2 to 18 | Original report-level citations not recoverable from the surviving screening record | Exact report-specific reasons unavailable. No retrospective reasons have been assigned. |
