# Supplementary material for: Adjunctive traditional Chinese medicine for ischemic stroke: a systematic review with structured narrative synthesis and GRADE assessment of randomized controlled trials
Source: Front Med (Lausanne). 2026 Sep 4;13:1884399. doi: 10.3389/fmed.2026.1884399 (PMC13585579; doi:10.3389/fmed.2026.1884399)
Supplement: Supplementary file 3 [file Table_2.docx]

**Supplementary Table 2. RoB 2 records for the specified result in each included trial**

The specified result is the effect of assignment to the intervention on each trial’s primary outcome. For the Xueshuan Xinmai trial, whose report designates no primary outcome, the specified result is the change in NIHSS at two weeks, selected as the most clinically relevant reported outcome. Overall judgments follow the RoB 2 algorithm and are never more favorable than the least favorable contributing domain. The judgments below are identical to Table 3 and Figure 3 of the main manuscript, to the GRADE profiles in Table 4, and to the Fig3_RoB2 worksheet of Supplementary Data File 1.

These judgments were reassessed during revision from published reports, available protocols, and registry records using the official RoB 2 signaling questions and algorithm. The surviving review records did not establish an independent duplicate reassessment, so the manuscript does not claim that the revised judgments were produced independently by two assessors.

| **Trial and specified result** | **Domain** | **Judgment** | **Rationale and supporting source information** |
| --- | --- | --- | --- |
| Tongxinluo (TISS)  mRS 0 to 1 at day 90 | D1 Randomization | Low | Randomization numbers generated independently by a statistician at the Peking University Clinical Institute using SAS version 9.4 via an automated centralized randomization system. Drug allocation was carried out independently by professional nurses. Baseline characteristics were balanced. |
|  | D2 Deviations | Low | A comprehensive blinding protocol was implemented. Placebo was matched to Tongxinluo in color, odor, taste, shape, texture, specification, appearance, packaging, labeling and identification. Analysis was by modified intention to treat. |
|  | D3 Missing data | Low | Outcome data were unavailable for 61 of 2007 randomized patients, so 1946 (96.5%) contributed to the primary analysis. Missing values were imputed by last observation carried forward. The proportion missing was small and balanced. |
|  | D4 Measurement | Low | mRS is a standard instrument assessed under double-blind conditions with a fully matched placebo. |
|  | D5 Selective reporting | Low | The study protocol and statistical analysis plan are publicly available with the published report. The reported primary outcome, mRS 0 to 1 at 90 days, corresponds to the planned primary outcome, prespecified subgroup analyses are identified as such, and all listed secondary outcomes are reported. The published abstract describes the design as both open-label and double-blind; the Methods describe comprehensive blinding in detail, so this is judged a typographical error in the abstract rather than an indication of selective reporting. |
|  | Overall | Low | All five domains judged low risk, so the algorithm yields low risk overall. |
| PANDA  mRS 0 to 2 at 3 months | D1 Randomization | Low | Randomization codes were generated using an automated centralized randomization system in a multicenter, double-blind, placebo-controlled trial. Baseline characteristics are reported as balanced between arms. The submitted version recorded this domain as some concerns; that judgment rested on an error about what the primary report describes and has been corrected here. |
|  | D2 Deviations | Low | Matched placebo at identical dose and schedule, double-blind throughout, analysis by modified intention to treat. |
|  | D3 Missing data | Low | 2966 of 3072 randomized patients (96.5%) were included in the modified intention-to-treat cohort. |
|  | D4 Measurement | Low | mRS assessed under double-blind conditions. |
|  | D5 Selective reporting | Low | Prospectively registered as ChiCTR1800016363. The trial protocol and statistical analysis plan are published as supplementary material with the primary report and were inspected during this revision. The published primary outcome, functional independence defined as mRS 0 to 2 at 3 months, is consistent with the protocol, the statistical analysis plan, and the registered outcome. The submitted version recorded this domain as some concerns on the basis of an error about the availability of these documents; that judgment has been corrected here. |
|  | Overall | Low | All five domains judged low risk, so the algorithm yields low risk overall. This replaces the some concerns judgment recorded in the submitted version. |
| ANGONG  Infarct and edema volume at day 14 | D1 Randomization | Low | Central stratified block randomization, 1:1, with blind-covered study medication assigned by serial number. Baseline characteristics comparable. |
|  | D2 Deviations | Some concerns | Three randomized participants never received study drug and were excluded from the full analysis set, so the analysis departs from the intention-to-treat principle. Eligibility also required a dialectical assessment by a traditional Chinese medicine physician, a subjective step whose timing relative to randomization could not be verified. |
|  | D3 Missing data | High | Of 120 randomized, 117 formed the full analysis set, and a further nine participants in the intervention arm and eight in the control arm did not undergo primary outcome assessment. Missing values were deleted in the primary efficacy analysis, making it a complete-case analysis with approximately one in six participants lacking the primary outcome. The report itself identifies the dropout rate as relatively high. |
|  | D4 Measurement | Low | Infarct and edema volumes were segmented centrally using automated software and assessed by radiologists blinded to allocation. An independent clinical event adjudication committee masked to assignment assessed outcomes. |
|  | D5 Selective reporting | Some concerns | Prospectively registered as NCT04475328 and both primary outcomes are reported. However the abstract and conclusions foreground a large-artery atherosclerosis subgroup result within a trial negative on both primary outcomes, and the report states that power and type I error were not calculated before initiation. |
|  | Overall | High | Driven by Domain 3. The overall judgment cannot be more favorable than the least favorable contributing domain. |
| CHIMES  mRS shift at 3 months | D1 Randomization | Low | International multicenter randomized, double-blind, placebo-controlled trial, prospectively registered as NCT00554723. |
|  | D2 Deviations | Low | Matched placebo over a three-month treatment period. No evidence of differential co-intervention between arms. |
|  | D3 Missing data | Some concerns | 1100 patients were randomized. A subsequent prognostic analysis of the same trial reports 1006 subjects with baseline data and a 3-month mRS, implying that approximately 8.5% lacked the outcome. The number contributing to the primary shift analysis and the method of handling missing data could not be confirmed from the primary report. |
|  | D4 Measurement | Low | mRS shift analysis conducted under double-blind conditions with a matched placebo. |
|  | D5 Selective reporting | Low | Prospectively registered. The published primary outcome, a shift in mRS at 3 months, corresponds to the registered primary outcome. Prespecified subgroup analyses are identified as such and the trial reports a null primary result with all secondary outcomes. |
|  | Overall | Some concerns | Driven by Domain 3. Under the RoB 2 algorithm the overall judgment cannot be more favorable than the least favorable contributing domain, so a trial with one domain raising some concerns cannot be judged low risk overall. The submitted version recorded this trial as low risk overall despite a Domain 3 judgment of some concerns, which was an algorithmic error and is corrected here. |
| Xueshuan Xinmai  NIHSS at 2 weeks | D1 Randomization | Some concerns | The report states that participants were randomly divided into two groups but does not describe sequence generation or allocation concealment. Registration is dated 9 November 2012 while enrollment ran from 1 January to 29 December 2012, so registration did not precede enrollment. |
|  | D2 Deviations | Some concerns | Placebo was matched in appearance, smell and taste and both arms received aspirin, atorvastatin and supportive care, but the analysis was not by intention to treat and the analytic model was not prespecified in any accessible document. |
|  | D3 Missing data | Some concerns | Two of 44 participants withdrew authorization, leaving 42 analyzed, and a further five were excluded from imaging analyses for head motion, so different analyses rest on different subsets. |
|  | D4 Measurement | Low | NIHSS and SSQOL are standard instruments applied by trained investigators under blinded conditions with a matched placebo. |
|  | D5 Selective reporting | High | Registration postdated the start of enrollment, so no prospectively registered outcome set exists against which to check reporting. The report designates no primary outcome. Clinical outcomes are reported only as group by time interaction terms, with no between-group effect estimate or confidence interval for any clinical outcome. |
|  | Overall | High | Driven by Domain 5. |
| Qizhitongluo  FMMS-LL at 12 weeks | D1 Randomization | Low | Prospectively registered as NCT01762163 in October 2013 before the study start. Central randomization in a 2:1:1 ratio across 13 sites. Baseline characteristics reported as comparable. |
|  | D2 Deviations | Low | Double-blind, placebo- and active-controlled, with aspirin and routine recovery training in all arms. Only the Qizhitongluo versus placebo comparison is assessed here. |
|  | D3 Missing data | Some concerns | The report does not state how many participants contributed to the week-12 primary outcome analysis or how missing data were handled, so the signaling questions for this domain cannot be answered from the available information. |
|  | D4 Measurement | Low | The Lower Limb Fugl-Meyer Motor Scale was administered by trained assessors under double-blind conditions. |
|  | D5 Selective reporting | Some concerns | The registry describes an adaptive design with a planned interim analysis after 312 participants completed treatment, but the adaptation rules and any resulting design changes are not described in the publication, and the protocol was not available. |
|  | Overall | Some concerns | Driven by Domains 3 and 5. |
| TIERS  Fugl-Meyer at 4 and 8 weeks | D1 Randomization | Some concerns | The report describes a double-blind placebo-controlled randomized phase II design but does not describe sequence generation or allocation concealment, and no trial registration was identified. |
|  | D2 Deviations | Some concerns | Placebo-controlled over four weeks in an inpatient rehabilitation setting, but the intensity of concurrent rehabilitation was not reported and could differ between arms. |
|  | D3 Missing data | Some concerns | Per-arm randomized and analyzed numbers are not stated beyond a total of 40 participants, so completeness of outcome data cannot be established. |
|  | D4 Measurement | Low | Fugl-Meyer Assessment, NIHSS and Functional Independence Measure applied under blinded conditions with a matched placebo. |
|  | D5 Selective reporting | Some concerns | No trial registration was identified, so no prospectively registered outcome set exists for comparison. Three instruments were measured at two timepoints, and the report presents subgroup tendencies alongside a null primary result. |
|  | Overall | Some concerns | Driven by Domains 1, 2, 3 and 5. |
| Naoxintong  Recurrence at 2 years | D1 Randomization | Low | Prospectively registered as NCT02334969. Participants randomly assigned 1:1 using a block design across 23 centers. |
|  | D2 Deviations | Low | Double-blind, placebo-controlled, analyzed using the intention-to-treat principle. |
|  | D3 Missing data | Some concerns | 301 of 2200 participants (13.7%) were lost to follow-up, 143 in the intervention arm and 158 in the control arm. The proportion is substantial for a time-to-event outcome, although the imbalance between arms is small. |
|  | D4 Measurement | Low | Recurrent ischemic stroke is an objective outcome assessed under double-blind conditions. |
|  | D5 Selective reporting | Some concerns | Prospectively registered and the primary outcome matches the registration, but secondary outcomes and safety events are reported without arm-level counts, and the protocol was not available for inspection. |
|  | Overall | Some concerns | Driven by Domains 3 and 5. |
| Dengzhan Shengmai  Stroke incidence at 1 year | D1 Randomization | Low | Registered as NCT00548223. Randomized, double-blind, placebo-controlled across 83 hospitals. |
|  | D2 Deviations | Low | Matched placebo, both arms receiving standard secondary prevention, analysis by intention to treat. |
|  | D3 Missing data | Some concerns | The published event counts and percentages (58, 3.8%; 82, 5.4%) imply per-arm denominators that do not sum to the 3143 randomized, so the number analyzed in each arm and the handling of missing data cannot be established from the report. |
|  | D4 Measurement | Low | Stroke recurrence is an objective outcome assessed under double-blind conditions with scheduled follow-up at 30, 90, 180 and 360 days. |
|  | D5 Selective reporting | Some concerns | The registered primary outcome of stroke recurrence at 1 year matches the publication, but the trial completed in January 2011 and was published in 2019, an interval of approximately eight years, and the protocol was not available. |
|  | Overall | Some concerns | Driven by Domains 3 and 5. |

Summary of overall judgments: two trials at low risk (Tongxinluo, PANDA), five raising some concerns (CHIMES, Qizhitongluo, TIERS, Naoxintong, Dengzhan Shengmai) and two at high risk (ANGONG, Xueshuan Xinmai).
